# Predictive simulation of coil mechanics uncovers shape-driven determinants of endovascular coiling efficacy in intracranial aneurysms

**DOI:** 10.1063/5.0312971

**Published:** 2026-08-04

**Authors:** Dario Tarantino, Alessandra Corvo, Chiara Livolsi, Lorenzo Nioi, Nicola Cuscino, Albert Comelli, Salvatore Pasta, Beatrice Bisighini

**Affiliations:** 1 Department of Engineering, Università degli Studi di Palermo, Viale delle Scienze Ed.8, Palermo, Italy; 2 Mines Saint-Etienne, INSERM, U 1059 Sainbiose, Centre CIS, F-42023 Saint-Etienne, France; 3 Department of Research, IRCCS ISMETT, via Tricomi, 5, Palermo, Italy; 4 Ri.MED Foundation, Via Bandiera, 11, Palermo 90133, Italy

## Abstract

Endovascular coiling is a minimally invasive therapeutic option for intracranial aneurysm (IA). This technique involves occluding the aneurysmal sac with one or more metallic coils to decrease perfusion of the dilated arterial wall. Although coil deployment has proven to be generally effective, clinical outcomes are difficult to predict, with aneurysms reopening occurring in approximately 20% of cases. The purpose of this study is to use *in silico* modeling combined with statistical analysis and machine learning to predict the biomechanical outcomes of coiling by studying the relationship between IA morphology and post-coiling quantitative parameters. First, a clinical database of IAs was parametrized based on a set of geometrical features. and 500 synthetic sac geometries were virtually generated. Simulations of coil deployment were then performed for each of these geometries, and the resulting reaction force, elastic energy, and contact pressure were used to train a predictive machine learning algorithm. The surrogate model demonstrated strong predictive performance for the reaction force (R^2^ = 0.74 and MAPE = 4.56%) and moderate performance for the elastic energy stored by the deformed coil (R^2^ = 0.68 and MAPE = 26.95%). Morphological factors such as aneurysm sac volume, surface, and height showed a good correlation with simulation parameters using Spearman analysis. As a preliminary proof-of-concept study, this methodology represents a first step toward the development of an *in silico* tool aimed at enhancing pre-operative planning for endovascular coil procedures. This has the potential to improve the stratification of patients at higher risk of complications and the identification of borderline IAs that may not be suitable for endovascular coiling.

## I. INTRODUCTION

An intracranial aneurysm (IA) is a focal dilation of a cerebral artery, characterized by an abnormal, localized, and persistent ballooning of its wall known as aneurysm sac.^1,2^ IAs commonly develop at the arterial bifurcations of the Circle of Willis.^3^ Although many IAs remain silent, their sac can enlarge due to blood inflow and potentially rupture, leading to a subarachnoid hemorrhage. This complication is associated with high rates of morbidity and is lethal in 45% of cases.^4,5^ Once an IA ruptures, prompt treatment is required to limit further bleeding. For unruptured aneurysms, similarly to aortic aneurysms, the IA dimension serves as the primary indicator of rupture risk and guides prophylactic intervention, with clinical guidelines recommending treatment for IAs greater than 7 mm in diameter.^6^ However, the size criterion alone is not considered a reliable prognostic factor of adverse events, as IAs smaller than 6–7 mm can also rupture.^7^

With advances in medical images and navigation systems, endovascular coiling has become the most widely used treatment technique for IAs.^8^ This endovascular procedure consists of introducing a flexible metallic coil into the aneurysm sac to prevent blood inflow and promote thrombus formation. For unruptured aneurysms, coiling helps reduce the hemodynamic pressure on the weakened arterial wall, thereby lowering the risk of rupture.^9,10^ Although clinical studies have reported that coiling achieves adequate IA occlusion in more than 90% of cases, aneurysm reoccurrence due to reopening occurs in approximately 20%, with aneurysms larger than 10 mm having a greater rate of failure.^11^ Potential risk factors for post-coiling aneurysm reopening are considered to be related to the final coil configuration, such as inadequate packing density and incomplete occlusion,^12^ as well as to the aneurysm morphology.^13^ In particular, IA morphology is commonly assessed through the neck-to-dome ratio, which is obtained by comparing the width of the aneurysmal neck—the orifice connecting the sac to the parent vessel—with the maximal dome diameter, corresponding to the point of greatest aneurysmal expansion. Nevertheless, the precise contribution of these factors to the risk of aneurysm reoccurrence remains poorly understood.^14^

In literature, several computational approaches have been proposed to model coil deployment within the IA geometry reconstructed from medical images.^15^ Among them, finite element simulations have been shown to realistically replicate coil packaging, including high-level coil modeling and patient-specific geometries.^14,16–19^ Other researchers focused on patient-specific simulations of the post-treatment hemodynamic.^20,21^ While extensive research has been conducted to investigate the relationship between aneurysm geometry and rupture risk from both clinical^22^ and computational perspectives,^23^ relatively limited research has focused on exploring the relationship between aneurysm morphology and the efficacy of coiling treatment.^24,25^

Recently, data-driven methods such as machine learning have been increasingly applied in biomechanics to integrate numerical modeling and identify hidden patterns between anatomical morphology and clinical outcome or mechanical performance with high accuracy.^26–31^ This integration enables real-time predictions for clinical scenarios where the use of traditional finite element modeling would be impractical due to the associated computational time. In this context, virtual geometries can be generated either through parametric approaches based on explicit geometric parameters^32^ or with non-parametric strategies, such as principal component analysis (PCA)-based statistical shape modeling. The latter allow complex anatomical variations to be captured and synthetic datasets to be generated, expanding beyond limited clinical data.^33^ While these techniques have been used to model other endovascular treatments, no study has yet investigated the application of such machine learning-based surrogate modeling to predict the outcomes of endovascular aneurysm coiling.

In this context, the current study aimed to develop a predictive tool that integrates finite element analysis with machine learning models to quantify and predict the mechanical performance of embolic coils in IAs with varying aneurysm morphology. The objective is to translate biomechanical knowledge into a rapid, clinically applicable tool to be used alongside current pre-operative tools like imaging analyses and manual measurements. We focused on the deployment of the first (framing) coil, which plays a critical role in establishing the initial coil scaffold and influencing the subsequent coil behavior.^34^ As a proof of concept for the application of machine learning to coiling simulations, we opted for a parametric generative approach over a non-parametric approach, as it provides systematic and independent control of each morphological parameter through explicit geometric formulations while also enhancing the interpretability of the results. To this end, we built an idealized parametric aneurysm model (Secs. V A and V B). Based on morphological ranges extracted from a multicenter clinical dataset of IAs, this model was then used to generate 500 idealized synthetic aneurysm geometries, which were employed in finite element coiling simulations (Sec. V C). Later, from the obtained simulations, we extracted key mechanical parameters at the end of the virtual procedure: the reaction force (RF), contact pressure (CPress), and strain energy (SE). This enabled us to obtain an extensive dataset of biomechanical indicators regarding the structural coil behavior with respect to aneurysm shape, with sufficient variability to train a machine learning model (Sec. V D). Specifically, a random forest regression with engineered features was built to predict biomechanical parameters using aneurysm geometry. In Secs. II and III, the results of the high-fidelity simulations and the machine learning models are presented and discussed, and Sec. IV offers some concluding remarks.

## II. RESULTS

### A. Biomechanical analysis

**FIG. 1.**
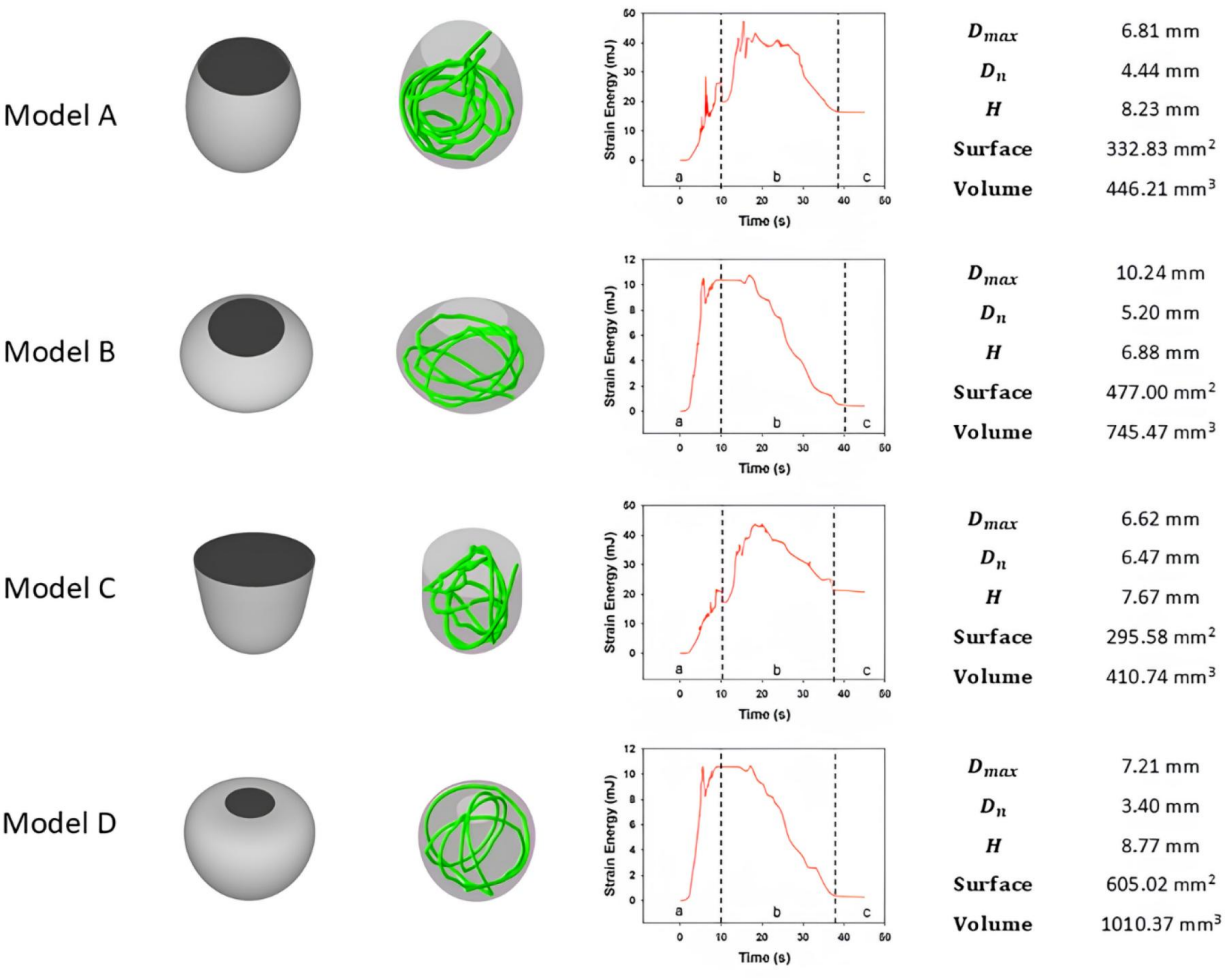
Four different aneurysms before and after coil deployment with their geometric characteristics. The plots represent the measured coil strain energy during the simulations: phase (a) corresponds to the initial packaging, phase (b) to the delivery of the coil inside the aneurysm sac, and phase (c) to the plateau where the coil is stabilized inside the aneurysm.

The entire database, including aneurysm geometrical descriptors and simulation output parameters, is publicly available in the project's GitHub repository (see Data Availability). Out of 500 simulations, none of the coil deployment in the IA sac failed due to numerical issues. As displayed in Fig. 1, the profile of the strain energy over the simulation time shows a steep increase during coil insertion in the catheter, followed by a peak region when the coil is fully inserted. Finally, a decrease toward a plateau region indicates a stable and stationary coil deployment into the aneurysm sac. Though the profile is common among different IA shapes, the magnitude of stored strain energy is remarkably influenced by the geometrical variability.

**FIG. 2.**
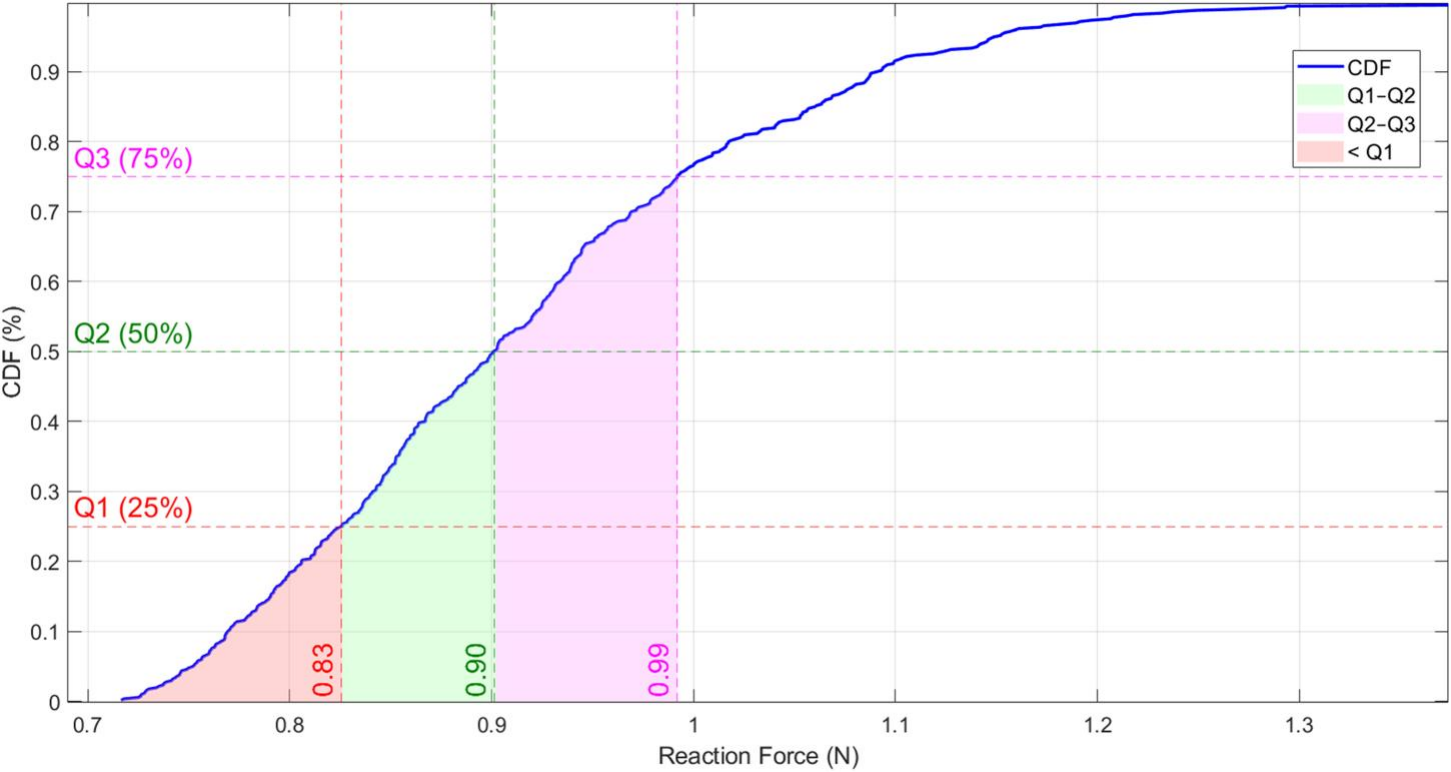
Cumulative density function (CFD) evaluating the reaction force (RF) variability across the 500 aneurysm morphologies. This function illustrates the probability of a randomly selected geometry requires an insertion force being below a specific threshold. The data are split into quartiles for qualitative visualization: Q1 (25%), Q2 (median, 50%), and Q3 (75%).

To assess the distribution of RF resulting from the simulations, a cumulative distribution function (CDF) was computed (Fig. 2). The CDF was obtained by aggregating the RF values from all 500 simulated aneurysm morphologies and represents the probability that the required insertion force is below a given threshold across the entire synthetic dataset. Quartiles were reported to facilitate interpretation. In particular, the median value (Q2) indicates that 50% of the simulated models required a reaction force lower than 0.9 N, while 75% of the cases required less than 1 N. Only 25% of the geometries exhibited higher mechanical resistance, requiring forces above 1 N for coil deployment.

**FIG. 3.**
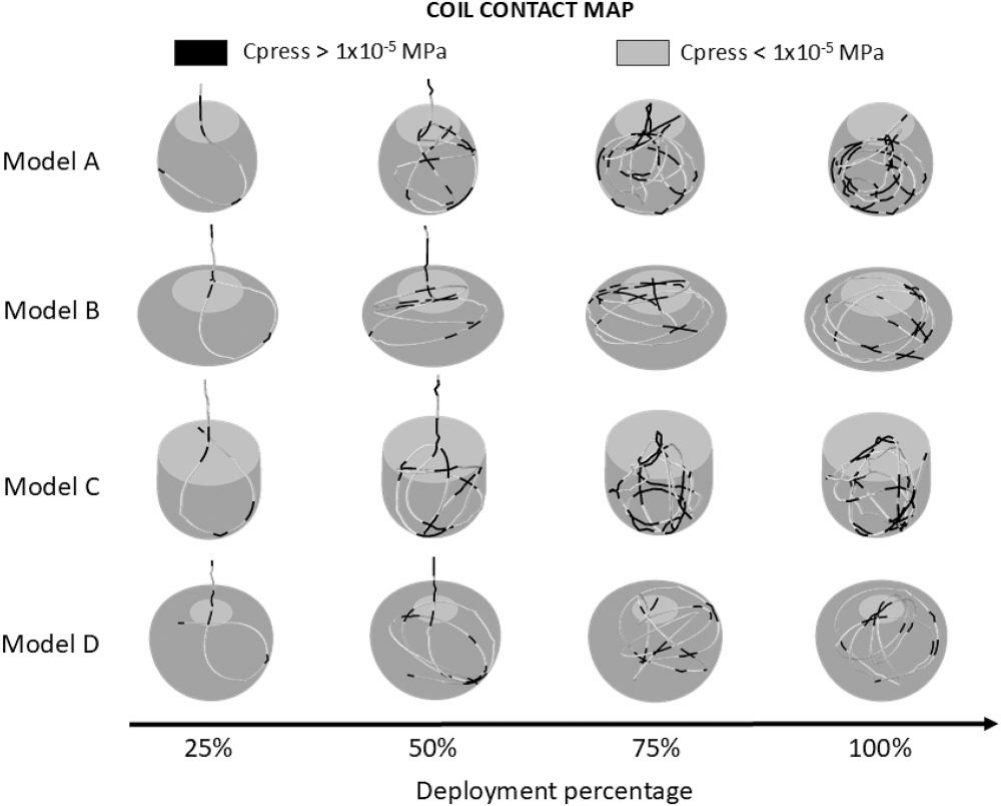
Distribution of the contact pressure (CPress) exerted by the coil on the aneurysm wall during deployment for four representative intracranial aneurysms geometries. Each row corresponds to a different aneurysm geometry, and the columns represent successive time frames during deployment. Black regions on the coil show areas where the local normal contact pressure exceeds 1 × 10^−5 ^MPa, highlighting the actual contact between the coil and the aneurysm. Light gray regions indicate no contact.

During the simulation, the greater the amount of the embolic coil filling in the sac, the higher the contact pressure between the coil and the aneurysm wall (Fig. 3). The maximum contact pressure was observed at the end of the simulation, with lower values likely associated with IA of larger diameter and height.

### B. Machine learning-based predictions and statistical analysis

**FIG. 4.**
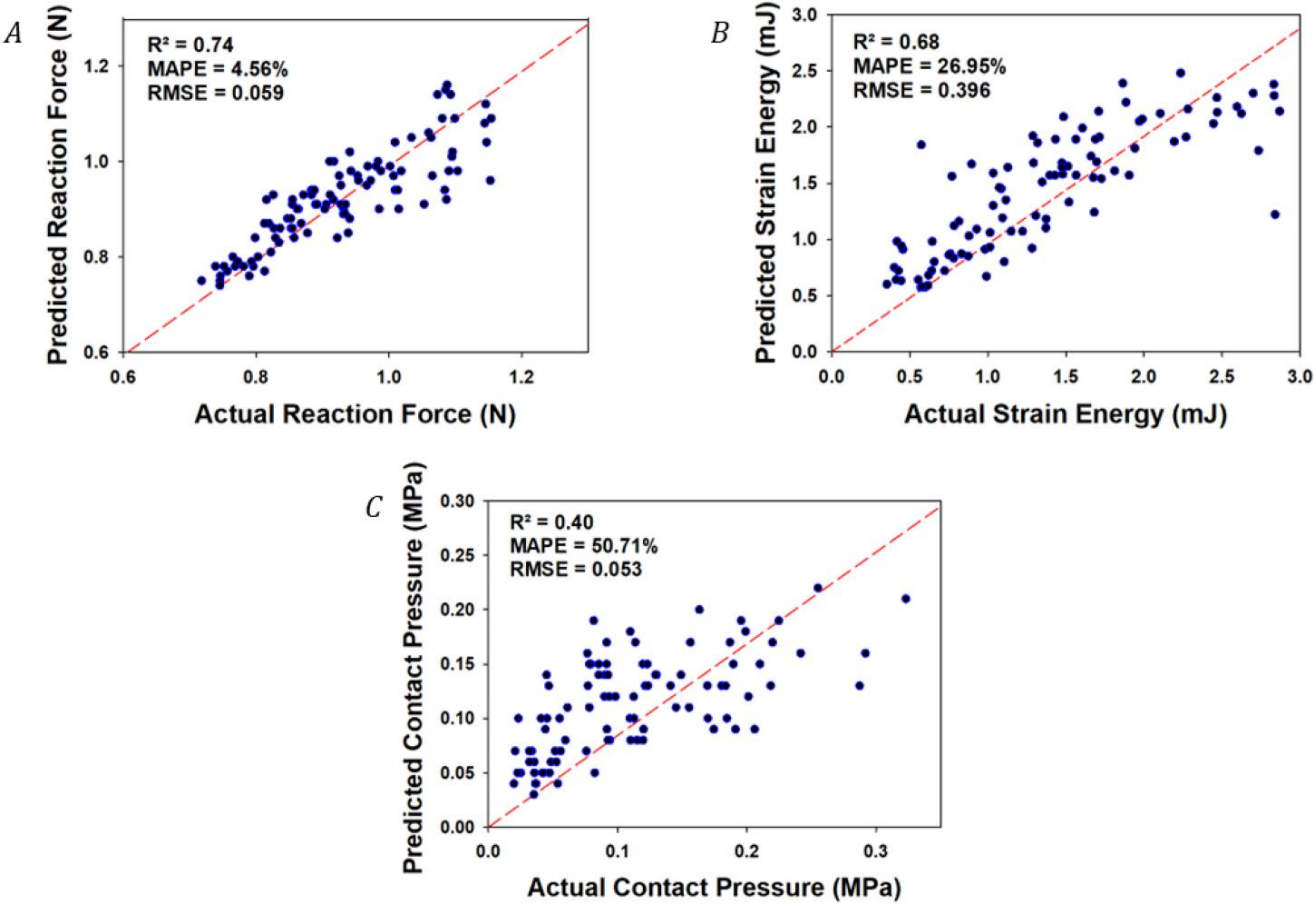
Performance of random forest regression models in predicting the biomechanical outcome: (a) reaction force, (b) strain energy, and (c) contact pressure. The x axis shows the actual values from the simulations, while the y axis shows the corresponding predicted values. Blue dots represent individual simulation data points, and the dashed red line represents the ideal surrogate model response, where predictions perfectly match the simulation results.

Random forest regression models were developed using second-order polynomial and interaction features to predict RF, CPress, and SE, with actual vs predicted values shown in Fig. 4. Regarding average RF over the simulation time, the model demonstrated strong predictive capability with an R^2^ of 0.74, a MAPE of 4.56%, and an RMSE of 0.059. For average SE, the model performance was slightly lower with an R^2^ of 0.68, a MAPE of 26.95%, and a RMSE of 0.396. Differently, the prediction of the CPress was low with an R^2^ of 0.40, a MAPE of 50.71%, and an RMSE of 0.053. The Lilliefors normality test indicated that both actual and predicted values for SE (p = 0.129 and 0.057) and RF (p = 0.188 and 0.746) are likely normally distributed. Furthermore, paired t-tests showed no statistically significant differences between predicted and actual values for energy (p = 0.192) and coil-related inserting force (p = 0.738), supporting the accuracy and reliability of the surrogate model for these biomechanical parameters. For the contact pressure, the Lilliefors test indicated that the actual values are not normally distributed (p = 0.007), while the predicted values are likely normally distributed (p = 0.376). Accordingly, a paired Wilcoxon signed-rank test revealed no statistically significant difference between the actual and predicted datasets (p = 0.518), suggesting that the surrogate model remains reliable despite deviations from normality in the actual simulation data.

**FIG. 5.**
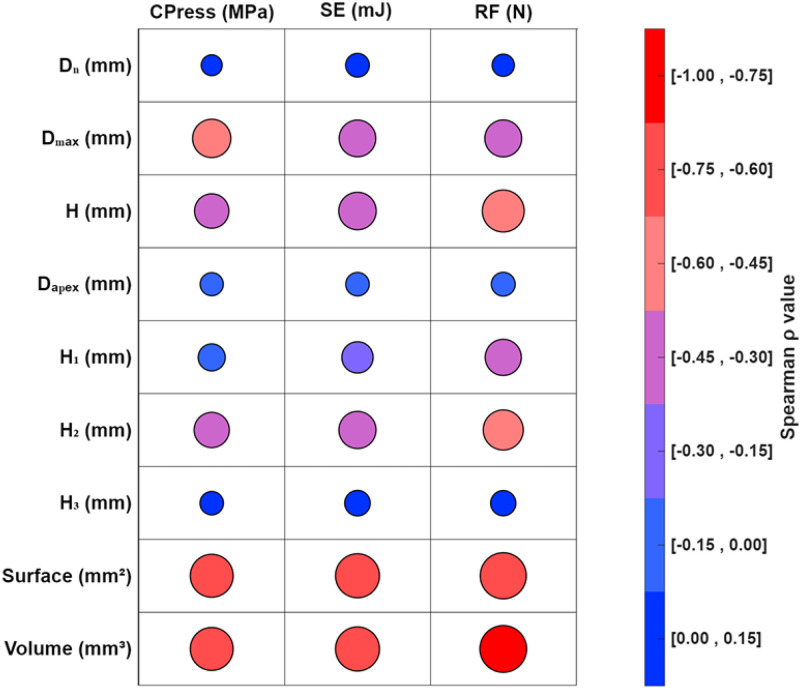
Correlogram illustrating the Spearman correlation coefficients between aneurysm geometrical input parameters and the predicted biomechanical simulation outcomes: contact pressure, strain energy, and reaction force. The color of the circles indicates the Spearman ρ value according to the color bar, while the size of the circles is proportional to the absolute strength of the correlation. All displayed correlations are considered statistically significant ( α < 0.05).

Among the IA morphological parameters, aneurysm volume and surface area demonstrated the highest correlations with the simulation output parameters predicted by the surrogate model, as shown by the correlogram in Fig. 5. Specifically, aneurysm volume had a good negative correlation with RF (R = −0.75) and SE (R = −0.65). Aneurysm surface area was associated negatively with the RF (R = −0.73) and SE (R = −0.65). Furthermore, aneurysm height (H) demonstrated a good negative correlation with RF (R = −0.58).

## III. DISCUSSION

This study examined the relationships between IA morphologies and structural parameters of the embolic coil procedure in a large synthetic cohort. Finite element modeling was employed to quantify key structural parameters, while machine learning techniques were used to develop fast predictive surrogate models of the simulation outcomes. To support this, a fully automated workflow was established to reduce the possibility of errors and enhance the overall efficiency of the study. Although the available literature is extremely limited, previous computational studies suggest that aneurysm morphology influences the final configuration of deployed coils and, consequently, the outcome of endovascular coiling.^22–25^ Holzberger *et al.*^25^ introduced a numerical framework for preliminary aneurysm occlusion classification; however, their analysis was limited to just three geometries, restricting the generalizability of their conclusions and preventing direct comparison with the present results. In contrast, our study employed an idealized parametric model of IA, which enabled a refined investigation of coil behavior across 500 geometrical configurations. This number of cases was selected in line with the authors' previous work on surrogate models and corresponding literature.^32^ The training dataset was generated using Latin Hypercube Sampling to ensure a uniform exploration of the geometric parameter space. This strategy ensured that the surrogate model was trained on a wide range of morphological configurations, including boundary cases that may be underrepresented in clinical datasets, thereby improving the robustness and generalization capability of the model across the explored design space.

Among the structural parameters of the coil simulation examined in this study, the plateau reached by the strain energy near the end of the insertion (Fig. 1), reflecting the stabilization of the coil within the aneurysmal sac, shows a remarkable variability across different IA shapes. This variability is mostly due to aneurysm surface and volume, as illustrated by the correlogram of Fig. 5: the larger the sac, the lower the strain energy encapsulated by the packaged coils. Similar results were observed for the average distribution of coil contact forces exerted on the aneurysm wall, with higher surface and volume values resulting in lower forces acting on the rigid arterial wall. This behavior is consistent with expectations: less dilated, and thus more compact, geometries provide greater resistance to coil release, resulting in higher contact pressure, making them more suitable for this type of treatment. Therefore, our findings suggest that aneurysm surface and volume, rather than conventional metrics such as aneurysm diameter, are critical determinants of embolic coil performance in the saccular IA. Because volume and surface area are closely correlated with other geometric descriptors, this analysis primarily reflects the effect of overall aneurysm size on coil performance rather than the impact of individual local features like the aneurysm diameter.

The reaction force at the coil proximal end is the most relevant clinical outcome of our finite element simulations, as this directly reflects the device resistance to the deployment process and provides a meaningful link between computational results and practitioners' maneuvers. Higher values indicate greater resistance during insertion, which could be due to more compact packing or effective mechanical engagement within the aneurysm sac. Using the cumulative distribution function (Fig. 2), we found that half of all simulations required less than 0.90 N of force to deploy the coil, representing the median behavior across morphologies between the 10th and 95th percentiles of the AneuX project. Only 25% of aneurysm models required more than 1 N of reaction force, implying that those aneurysm shapes may have less favorable geometries that cause more mechanical resistance during coil deployment. To demonstrate the reliability of our coiling simulations, a direct comparison with experimental measurements performed by Matsubara *et al.*^35^ was performed. In their study, an optical sensor was mounted on the catheter surface to measure coil insertion, finding an average RF of 0.7 ± 0.5 N across ten different IAs with sizes ranging from 7.4 to 14.2 mm and coil diameters of 6 mm. Our computational model predicted an RF of 0.85 ± 0.3 N for aneurysms in the same size range. This close agreement between numerical predictions and experimental data strengthens confidence in the computational outcomes. It also supports the assumption of a rigid aneurysm wall in our simulations. However, as stated in the limitations section of Matsubara *et al.'s*^35^ study, their sensor system measures the coil insertion force only at the proximal portion of the microcatheter. Therefore, the forces reported by Matsubara and collaborators include catheter-related friction effects. Consequently, the agreement between our numerical predictions and their experimental data should be interpreted strictly as a plausibility check rather than as a validation of the coil constitutive model.

Surrogate modeling is emerging as a solution for machine learning-enabled real-time predictions in the context of medical device simulation, which may overcome the significant computational costs and technical expertise required for developing a computational model.^29,30,36^ Here, we investigated the potential of surrogate modeling to predict key structural parameters of embolic coils. The approach could lead to a tool capable of predicting coil outcomes based solely on geometrical characteristics of the candidate patient's aneurysm as determined by routine pre-operative imaging. As shown in Fig. 4, the average RF-related prediction had a MAPE of 4.56%, indicating that the surrogate model's predictions deviated approximately 5% from the simulated coil-related forces. Comparable prediction errors have been reported in previous studies,^28,29,36^ suggesting the reliability of this methodology. Contact pressure was the most difficult variable for surrogate-related regression, which resulted in significantly lower performance. This limitation can be attributed to the local nature of contact pressure, which may be influenced by geometric details and the rigid wall approximation used in the model.

In conclusion, this work provided insight into the design of risk classification strategies for embolic coiling that go beyond purely image-driven criteria. The most notable finding is the capacity of the surrogate model to rapidly and accurately predict structural output characteristics of the implanted coil in relation to the morphological variability of aneurysm sacs. Surface area and volume emerged as the dominant predictors of coil behavior, underscoring the importance of overall aneurysm dimensions in determining coil performance. While providing improved biomechanical insights into coiling, the present study presents some limitations that should be addressed in future research. First, vessel walls were assumed to be rigid with uniform wall thickness. While this simplification is commonly adopted in the literature due to the lack of precise patient-specific wall properties, it does not fully capture the biomechanical behavior of real aneurysms and could have an impact on the computed biomechanical metrics. Second, the analysis was restricted to the framing phase of coiling, naming the insertion of the first coil. In clinical practice, multiple coils are typically deployed to achieve more complete occlusion, thus future studies should incorporate this aspect in the simulations. Nevertheless, the framing phase is generally considered the most critical step of the procedure, as it mainly determines treatment output.^37^ Another limitation concerns the mechanical representativeness of the adopted coil model. The present work is intended as a proof-of-concept study and employs a simplified, standardized solid-beam representation of the embolization coil, whose quantitative agreement with the mechanical behavior of actual devices remains uncertain. To preliminarily assess the influence of beam cross-sectional stiffness, an additional sensitivity analysis was performed using a hollow circular beam section with an outer diameter of 0.30 mm and an inner diameter of 0.20 mm. This pipe-like representation reduced the predicted reaction force by approximately 26% compared with the reference model, confirming that the simulation outputs are sensitive to the assumed beam stiffness. However, this analysis does not fully address the underlying mechanical discrepancy. In homogenized coil models, the effective bending stiffness arises from the helical microstructure of the device and depends strongly on the stock-wire geometry, scaling approximately with the fourth power of the stock-wire diameter. This characteristic scaling is not captured by the present beam formulation, and the effective stiffness of actual embolization coils may therefore differ substantially from that assumed in the simulations. Accordingly, the absolute values of reaction force, strain energy, and contact pressure reported in this study should be interpreted with caution. The objective of this work is not to provide quantitatively validated predictions of coil mechanics but rather to investigate morphology-dependent relative trends using a standardized coil representation.

Finally, despite being derived from a clinical database, the current parametrized aneurysm model still represents an idealized geometry. More realistic parameters could be incorporated in future studies.^38,39^ Future work will focus on developing a metric to select coils based on aneurysm morphology, reflecting typical clinical decision-making, to enhance the framework's generalizability. Moreover, we plan to incorporate additional parameters more directly related to aneurysm rupture risk during the procedure, addressing the limitation that the coil proximal tip reaction force may not capture peak local wall loading when the coil bends laterally or load paths become non-axial. Specifically, calculating the maximum reaction force along the coil may provide a more clinically relevant metric for assessing the risk of aneurysm wall perforation caused by microcatheter manipulation. Furthermore, future work will aim to introduce more realistic aneurysm and coil models, including deformable vessel walls and a homogenized effective stiffness that accounts for the actual helical structure of the coil, in order to provide a more physiologically accurate estimation of the biomechanical metrics under interest.

## IV. CONCLUSION

This study demonstrated the effectiveness of a fully automated and robust workflow for coiling simulation in idealized synthetic IA geometries. The findings revealed a physical link between aneurysm morphology and coil deployment, offering new insights into the understanding of endovascular occlusion processes. The integration of machine learning models further enabled rapid and reliable estimations of the structural performance of coil simulations, reinforcing the potential clinical impact of this approach. Future studies will focus on evaluating the generalizability of the proposed surrogate model on patient-specific IA geometries to assess how training on idealized synthetic datasets translates to real anatomical variability and clinical scenarios.

## V. METHODS

### A. Database analysis

Seven fifty shapes were extracted from the AneuX project (2015–2020), an open-access, multi-centric clinical database of 3D IA geometries, in which the aneurysm sac had already been isolated from the parent artery.^40^ We investigated the IA sacs with a height-to-neck diameter ratio ranging from 1.1 to 1.6. Aneurysms with atypical morphology or excessively large necks were excluded, as these are typically not treated with coiling alone but instead require adjunctive devices, such as balloon-assisted or stent-assisted coiling, or with flow-diverter devices.^41^ These exclusion criteria were designed to eliminate complex morphologies, thus ensuring a controlled and standardized reference base for generating the synthetic dataset. These thresholds were also selected to include only aneurysm geometries compatible with the coil sizes used in the simulations. A final database of 132 cerebral aneurysm models was obtained, from which geometrical parameters, including (i) the maximum diameter ( $D_{\text{max}}$), (ii) neck diameter ( $D_{n}$), and (iii) aneurysm height ( H), were measured. Only values between the 10th and 90th percentiles were retained for these parameters, defining acceptance bounds that excluded statistical outliers and ensured physiologically plausible geometries during the subsequent generation of synthetic geometries.

### B. Parametric model

**FIG. 6.**
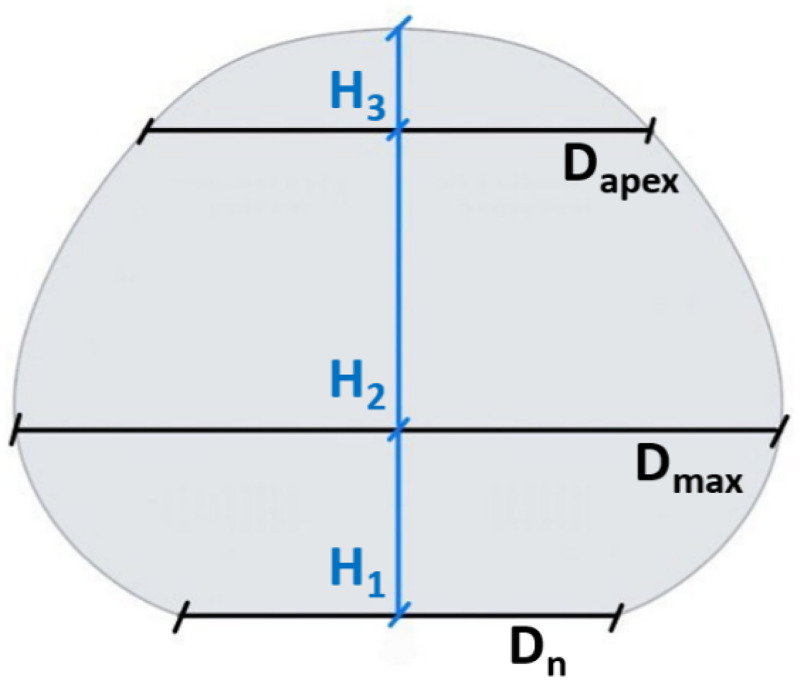
Schematic representation of the idealized model of intracranial aneurysm and definition of the geometric parameters used for shape variability quantification. The parametric model adopted seven variables to generate synthetic dome shapes, including three independent geometric parameters, three dependent height values, and one additional diameter value.

A framework for designing a large database of idealized IA models was developed using the Grasshopper application in the computer-aided-design (CAD) software Rhinoceros 3D (v7, Robert McNeel & Associates, USA). To capture the aneurysm shape variability, seven parameters were adopted to generate the dome-like aneurysm shapes: three independent geometrical parameters ( $D_{\text{max}}$, $D_{n}$, and H), three dependent height values ( $H_{1}$, $H_{2}$, and $H_{3}$) and one additional diameter value ( $D_{\text{apex}}$), as shown by Fig. 6. The different heights were computed as follows:

$$\begin{array}{c} H_{1}=B\cdot H_{2},\\ H_{2}=H/(A+B+1),\\ H_{3}=A\cdot H_{2}, \end{array}$$ (1)

where $H_{1}+H_{2}+H_{3}=H$ and A and B are two morphological parameters selected as 0.1≤A≤0.9 and 0.4≤B≤0.9. This equation was introduced to divide the total aneurysm height intro three segments defined by the three diameters ( $D_{\text{max}}$, $D_{n}$, and $D_{\text{apex}}$) and the selected ranges for A and B were chosen empirically to ensure that the generated intracranial aneurysm shapes remain physiologically realistic, avoiding any concavity in the generated geometry. Surface and volume of IA models were also computed. Table I summarizes the ranges of all morphological parameters.

**TABLE I.** Range of geometrical parameter values used for generating the aneurysm shape database (^*^ indicates a parameter obtained from the clinical database).

Parameter	Range
$D_{\text{max}}\;\left(\text{mm}\right)$ ^*^	5.58–10.25
$D_{n}\;(\text{mm})$ ^*^	3.25–6.48
$D_{\text{apex}}\;(\text{mm})$	5.58–6.48
H (mm) ^*^	4.17–8.78
$H_{1}\;(\text{mm})$	0.0038–3.88
$H_{2}\;(\text{mm})$	1.59–7.55
$H_{3}\;(\text{mm})$	0.22–3.50

From this parametrized model, a synthetic dataset of 500 aneurysm geometries was generated by sampling the geometrical parameter ranges indicated in Table I using the Latin Hypercube Sampling (LHS) technique in Matlab (v2023, MathWorks, USA). Automatic scripting was implemented to generate and export each IA model as a stereolithography file (STL) from Grasshopper. Since the parameter ranges had already been restricted as described in Sec. V A, no additional acceptance criteria were required, and all generated geometries were deemed suitable for simulation following visual inspection.

### C. Finite element analysis

**FIG. 7.**
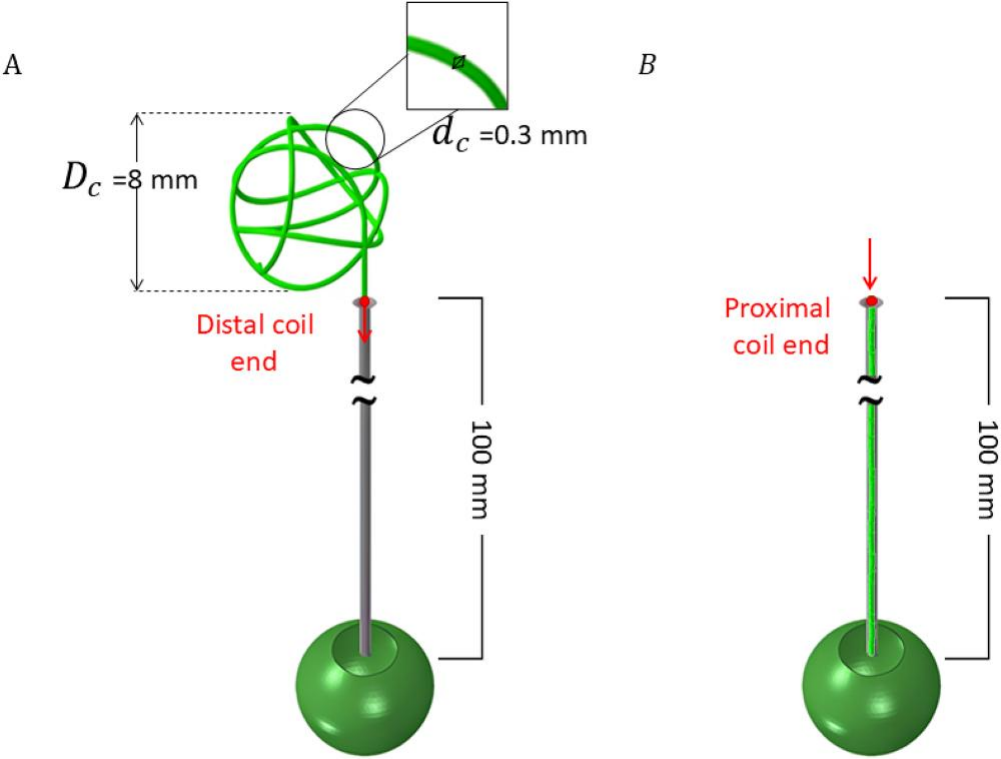
Setup for the finite element simulation of the endovascular coiling procedure. (a) Reference configuration of the model, showing the aneurysm sac, the catheter, and the coil generated at the free configuration. (b) Detail of the end of the packaging phase, in which the coil is fully inserted inside the rigid catheter, just before deployment. Red markers indicate the nodes where boundary conditions are imposed: at the distal coil end during the packaging phase (a) and at the proximal coil end during the deployment phase (b).

The simulation of coiling consists of three components: the aneurysm sac, the coil, and the catheter. The geometries of the aneurysms were meshed with triangular elements (S3) with an element size of 0.3 mm. As previously done in Refs. 14 and 16, the aneurysm was modeled assuming rigid wall condition. As proposed in Ref. 14, we modeled the coil as a thin wire with a filled cross section with diameter $d_{c}=0.3\text{ mm}$. As shown in Fig. 7(a), a “complex” free configuration with diameter $D_{c}=8\text{ mm}$ and length of 100 mm was generated using parametric equations implemented in a custom Matlab script. The obtained geometry was discretized using Timoshenko beam elements (B31). This choice of a complex-shaped coil with 8 mm diameter and 100 mm length reflects current clinical practice, as such coils are commonly used in the framing stage to provide a stable initial scaffold,^42^ achieve realistic packing behavior, and are appropriate for aneurysms of small to medium size.^43^

The metallic wire (92% platinum and 8% tungsten) was modeled using a linear-elastic material with a Young's modulus of 7.5 GPa, a density of $21.3\;g/{\text{cm}}^{3}$, and a Poisson's coefficient of 0.39.^16^ A mesh sensitivity analysis was performed to evaluate the robustness of the predictions and identify the optimal element length. The analysis compared a baseline of 200 beam elements (0.50 mm length) against coarser discretization of 185 (0.54 mm) and 150 (0.67 mm) elements. The quantitative results of this sensitivity assessment are summarized in Table II. While coarser mesh discretization resulted in only moderate deviations in global response quantities (up to ∼19% for reaction force), they failed to accurately capture highly sensitive local metrics, with contact pressure deviations reaching as high as ∼81%. In contrast, further mesh refinement led to numerical instabilities that compromised the robustness of the simulations. Therefore, a mesh resolution of 0.5 mm was identified as the optimal mesh size, providing an effective balance between accurately resolving complex local contact mechanics and maintaining numerical stability. Moreover, this resolution is consistent with values reported in the literature for comparable deployment models (∼0.3–0.5 mm).^14,16^

**TABLE II.** Mesh sensitivity analysis showing the dependence of global and local simulation outputs on coil discretization.

Beam number	Beam length (mm)	Average SE (mJ)	Average RF (N)	Average CPress (MPa)	Error SE (%)	Error RF (%)	Error CPress (%)
200	0.50	8.597	0.927	0.843	0.00	0.00	0.00
185	0.54	7.878	1.029	0.413	8.37	10.98	51.00
150	0.67	6.974	1.103	0.160	18.89	19.04	81.08

The catheter was modeled as a rigid tube with a diameter of 0.6 mm and length equal to 100 mm (same length as the coil). The proximal end of the catheter was blunted to facilitate coil access. The coiling procedure was simulated using the finite element solver Abaqus/Explicit (v2021, Simulia, USA). The simulation process involves first coil packaging, followed by its release in the IA. During the packaging phase, the coil was pulled into the catheter by aligning its distal end with the catheter tip. A vertical displacement boundary condition, equal to the length of the coil, was applied to the distal coil end. Frictionless contact conditions were enabled between the catheter and the coil outer surface. A dummy step of 5 s was added after the packaging step to reduce undesired dynamic oscillations before the release. Then, the deployment was simulated by pushing the metallic wire into the IA wall, applying a vertical displacement boundary condition, equal to the length of the coil, to the proximal coil end. As suggested in other studies,^14,18,19^ a penalty contact with a coefficient of 0.2 was adopted to account for the interaction between the IA wall and the coil. A quasi-static approach using mass scaling was used for all simulation steps with numerical stability ensured by keeping kinetic energy below 10% of internal energy. Simulations were carried out using 8 nodes of a computing cluster equipped with 112 CPUs (Intel Xeon Gold 6132) for a total time of 2 days for the whole database.

After each simulation, automatic scripting was adopted to extract quantitative data on coil performance to reduce the risk of user errors and speed up post-processing. The simulation outputs included the reaction force (RF) measured at the proximal coil end, contact pressure (CPress), and coil strain energy (SE) of embolic coils. Specifically, RF reflects the coil-induced forces during deployment into the aneurysm sac. It captures the practitioner's experience of the handling force and represents an indicator of insertion difficulty. CPress quantifies the local normal force per unit area at the interface between the coil and the aneurysm wall and, thus, the anchoring force of the device within the surrounding tissue. Finally, SE represents the elastic potential energy stored in the coil wire at the end of the simulation, providing insight into the energy distribution resulting from coil deformation.

### D. Surrogate modeling and statistical analysis

Prior to machine learning, a total of 24 outliers from the simulation outputs and corresponding geometrical features were removed using the interquartile range (IQR) criterion, excluding values above 75th percentile+1.5×IQR (with IQR=75th−25th percentile). Then, second-order polynomial and interaction features were computed from the geometrical parameters, normalized using min–max scaling, and subsequently used to train a random forest regression model implemented in Python (v 3.11.0) using *pandas* (v2.2.3) and *scikit-learn* (v1.5.2). The random forest regression model is an ensemble learning algorithm that constructs multiple decision trees using bootstrapped samples of the data and averages their outputs to improve predictive accuracy and reduce overfitting.^44^ In this study, the main hyperparameters were set as follows: the number of estimators was 1000 (i.e., 1000 trees in the forest) and the random state was set at 2 to ensure reproducible results. All other hyperparameters used the default scikit-learn settings.^45^ These settings allow each tree to grow fully without predefined depth limitations while using all available features at each split, thereby enabling the model to flexibly adapt to data complexity. This surrogate model was developed to predict the simulation output parameters, RF, CPress, and SE, from the IA geometrical descriptors. The dataset was split into 80% for training and 20% for testing. Model performances were quantified using the coefficient of correlation (R^2^), mean absolute percentage error (MAPE), and root mean squared error (RMSE). Moreover, statistical analysis was carried out to compare actual vs predicted simulation outputs in the test set. After normality assessment using Lilliefors normality test, a paired t-test or Wilcoxon signed-rank test was performed to evaluate potential statistical differences between the actual and predicted simulation output.

Parallelly, a Spearman correlation analysis was carried out to explore the relationship between biomechanical and aneurysm geometrical parameters, removing outliers from both groups and normalizing the values using min–max scaling. Statistical significance was considered for α≤0.05.

## Data Availability

The data that support the findings of this study are openly available in github at https://github.com/DarioT428/EndovascularCoiling.git, Ref. 46.
